# Dendrimer Coated Silica as a Sorbent for Dispersive Solid-Phase Extraction of Select Non-Steroidal Anti-Inflammatory Drugs from Water

**DOI:** 10.3390/molecules29020380

**Published:** 2024-01-12

**Authors:** Piotr Ścigalski, Przemysław Kosobucki

**Affiliations:** Department of Food Analysis and Environmental Protection, Faculty of Chemical Technology and Engineering, Bydgoszcz University of Science and Technology, Seminaryjna 3, 85-326 Bydgoszcz, Poland; p.kosobucki@pbs.edu.pl

**Keywords:** liquid chromatography, solid-phase extraction, environmental protection, trace analysis, pharmaceutical contamination

## Abstract

Non-steroidal anti-inflammatory drugs (NSAIDs) have been recognized as a potentially serious threat to the natural environment. NSAIDs are popular painkillers, and the main pathway for them to reach natural water is via discharge from wastewater and sewage treatment plants. In order to monitor contamination caused by these drugs, as well as their impact on the environment, a new material based on Silica Gel 60, functionalized with a dendrimeric copolymer of methylamine and 1,4-butanediol diglycidyl ether (named MA-BDDE), was prepared. Initial physicochemical characterization of the MA-BDDE material was carried out using ATR FT-IR spectroscopy as well as solid-state carbon-13 NMR spectroscopy. Its effectiveness at NSAID extraction was evaluated by the application of five select drugs in dispersive solid-phase extraction (dSPE): aspirin, ketoprofen, naproxen, diclofenac and ibuprofen. This was followed by their simultaneous determination using the HPLC-UV/Vis system demonstrating good sensitivity, with limits of detection values within the 63–265 ng mL^−1^ range. A comparison of the sorption capacity of each pharmaceutical with unmodified base silica showed an at least tenfold increase in capacity after modification. Initial MA-BDDE application in a quick, low-waste extraction procedure of those select NSAIDs from spiked surface water samples yielded promising results for its use as a sorbent, as recovery values of analytes adsorbed from various samples were found to exceed 72%.

## 1. Introduction

Civilizational advances can often have side effects that negatively impact the natural environment in several ways, one of which is the introduction of new pollutants into natural water resources. The increasing world population and the drive to improve the standard of living around the globe, resulting in constantly growing industrialization and agriculture, are the major reasons behind declining water conditions [1,2].

Pharmaceutical contaminants, including non-steroidal anti-inflammatory drugs (NSAIDs), while playing an important role in the effort to protect human health, have recently been recognized as a growing threat to the natural environment [3,4]. Their trace amounts, as well as those of their metabolites and the products of their biodegradation (hydrolysis and photolysis), have recently been detected in samples of surface and ground natural water and soil [5,6]. Across the world, studies have been conducted within the last decade in order to determine the concentrations of pharmaceuticals in rivers. European rivers were shown to have the lowest NSAID contamination levels. In some regions, particularly those where the employed water treatment technologies were not of the highest quality or did not provide sufficient processing capacity, concentrations of certain drugs in the water were reported to be as high as 100 ng mL^−1^ [7,8,9,10].

The following urge to more closely monitor their presence in the environment leads to the identification of main entry pathways responsible for releasing the highest loads of NSAIDs and their derivatives. Due to their incomplete metabolism, some drug amounts are excreted through defecation and urinary systems and remain unchanged. These pharmaceutical residues reach sewage and wastewater treatment plants, yet employed treatment techniques often are not appropriate or efficient enough to treat concentrations present in sewage from hospitals and residential areas. As a result, portions of these drugs pass through wastewater treatment plants unprocessed and are released directly into rivers. Additionally, discharge of only partially treated or untreated effluents from pharmaceutical industries into open areas and streams, as well as the illegal disposal of unused (i.e., expired) drugs, further contributes to the contamination of the environment [11,12,13].

Non-steroidal anti-inflammatory drugs are a class of therapeutic drugs whose main function is the inhibition of cyclooxygenase enzyme activity responsible for the synthesis of biological mediators involved in inflammation and blood clotting. As such, they are commonly used to prevent blood clotting and combat fever but have also found wide applications as effective painkillers. The effectiveness and wide applicability of NSAIDs, together with their low cost and high availability (often without prescription), make them a preferred medicine of choice in many hospitals and households [6,12]. As a result of NSAID consumption, their demand and production have steadily increased, resulting in higher loads reaching wastewater and sewage treatment plants and, ultimately, the environment. This growing concern recently caused the European Union to re-evaluate its approach to water contamination, as detailed in a communication from the European Commission to the European Parliament and the European Council [14], advising the improvement of environmental monitoring and the assessment of risk posed by pharmaceuticals.

Many accurate analytical methods already developed for this purpose consist of a chemical species separation process. Most commonly used techniques are usually based on chromatographic procedures such as gas chromatography (GC) or liquid chromatography (LC); the latter is now mostly employed in its high-performance iterations, known as HPLC (high-pressure liquid chromatography) and UHPLC (ultrahigh-pressure liquid chromatography), as well as electrophoretic techniques such as capillary zone electrophoresis (CZE). Target quantification in the sample is carried out based on the analyte type using one or more (out of many) diverse detection techniques, which, among others, include atomic emission (AES) and absorption spectroscopy (AAS), in the case of metal ions, and mass spectrometry (MS), ultraviolet (UV), fluorescence (FL) or visual light (Vis) spectrophotometry for organic and inorganic compound determination.

Environmental samples arriving at a laboratory usually need treatment aimed at separating the target analyte due to large amounts of particulate and chemical interferents that render them unsuitable for direct analysis [15,16]. Samples exhibiting more complex matrices require the employment of complicated procedures often containing multiple steps in order to perform accurate analysis, turning the sample pre-treatment into the bottleneck of the entire process [17,18]. As a result, laboratories around the world conduct research in order to find new materials and methods, allowing for more effective, rapid, and green sample clean-up, or to improve upon existing solutions.

Liquid–liquid extraction (LLE) is one of the oldest pre-concentration methods still in wide use, in which mixing a liquid sample with immiscible solvent results in the transfer of the analyte from the sample into the volume of the solvent. This technique is time-consuming and labor-intensive while also requiring large volumes of organic solvents, making it expensive and impractical in application. In more modern solid-phase extraction (SPE), the volume of the sample comes into contact with a solid sorbent material designed to have a strong physical or chemical affinity towards the target analyte, resulting in its adsorption onto the solid surface. SPE is considered to exhibit several crucial advantages over LLE: it requires lower amounts of solvents, time and labor while providing higher effectiveness and selectivity. Additionally, this technique is much easier to automate and integrate with other pre-treatment and analytical procedures, leading to the development of numerous modifications and combinations, such as solid-phase microextraction (SPME), stir bar sorptive extraction (SBSE), matrix solid-phase dispersion (MSPD) or ion-exchange solid-phase extraction (IE-SPE). Solid-phase extraction and its many iterations have now become one of the most widely used sample preparation techniques for both solid and liquid samples [15,16,17,18].

In 2003, Anastassiades et al. [19] presented an extremely simple extraction procedure used as a clean-up step in the analysis of pesticide content in agricultural products called dispersive solid-phase extraction (dSPE), where a powdery sorbent was applied directly into the volume of the sample solution and stirred. This allowed quick acquisition of the extraction equilibrium due to the high contact surface between the sorbent and the sample, while requiring only small amounts of solvent. dSPE was therefore introduced as a method that is simple, rapid and effective and generates little waste, resulting in its quick and wide recognition, becoming the base for QuEChERS multiresidue analysis [18,20].

Currently, one of the materials most widely used as a sorbent is silica, typically applied in normal phase procedures based on physical adsorption [16], despite its limited pH tolerance and lack of surface functional groups, resulting in only a singular sorption mechanism, which severely limits possible applications. It is, however, a material that is rigid and easy to synthesize, exhibiting resistance to shrinking and swelling, and the ease with which it can be modified offsets its disadvantages to a significant degree. In addition to this, mesoporous silica exhibits an organized internal structure containing uniformly distributed mesopores, assuring a significantly higher surface area [21,22].

Polymers based on crosslinking organic resins are among the materials most commonly used to modify silica. Their particular properties directly depend on the monomers used for synthesis and parameters of the polymerization reaction, allowing for high reliability of the process and precise control over the final product. Aside from the potential for high surface areas, they offer significant advantages over other materials, such as rich functional diversity and high chemical stability, especially against water. Considering some polymers show relatively weak physical properties that would be offset by immobilizing them on a silica core, both materials complement each other’s strengths and weaknesses perfectly, promising a hybrid material of much wider applicability than its constituents [16,23,24].

Among polymers, dendrimers are a unique type of macromolecule carrying highly complex, branched, three-dimensional internal structures with an external surface exhibiting remarkably high functionality. Like most polymers, dendrimer-specific properties are dependent on various combinations of diverse components available for synthesis, as well as on parameters by which the reaction was carried out. It is, therefore, entirely possible to reliably yield a product designed to offer highly specific characteristics such as particle size, chelation ability, stability, etc. [25,26].

This study presents a new porous material consisting of a silica core coated with a dendrimeric copolymer based on methylamine (MA) and 1,4-butanediol diglycidyl ether (BDDE). A quick, low-waste analytical procedure consisting of dSPE NSAID isolation and HPLC-UV/Vis detection was developed and utilized to evaluate the obtained materials’ (labeled as MA-BDDE) sorption effectiveness from spiked surface water samples.

## 2. Results

### 2.1. Sorbent Characterization

**Figure 1:**
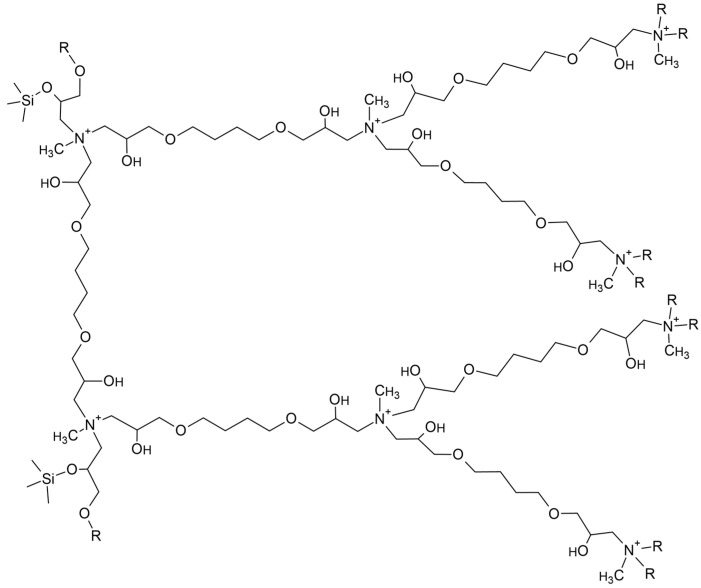
Hypothetical internal structure of the MA-BDDE material.

For the purpose of isolating NSAIDs from environmental surface water samples, a hybrid sorbent, named MA-BDDE and consisting of a silica core layered with a dendrimeric copolymer of methylamine and 1,4-butanediol diglycidyl ether, was prepared as described in Section 4. Figure 1 illustrates part of the expected macromolecular structure of the material, showing a binding between the polymer and the silica core, and the first two dendrimeric layers.

**Figure 2:**
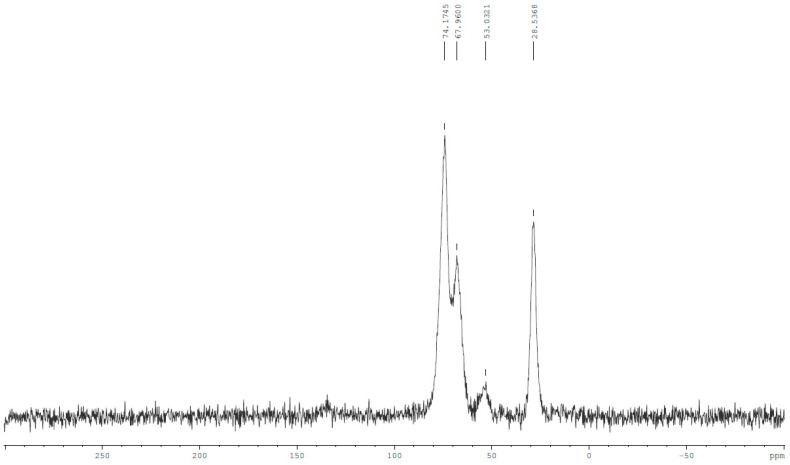
NMR spectrum of MA-BDDE material.

**Figure 3:**
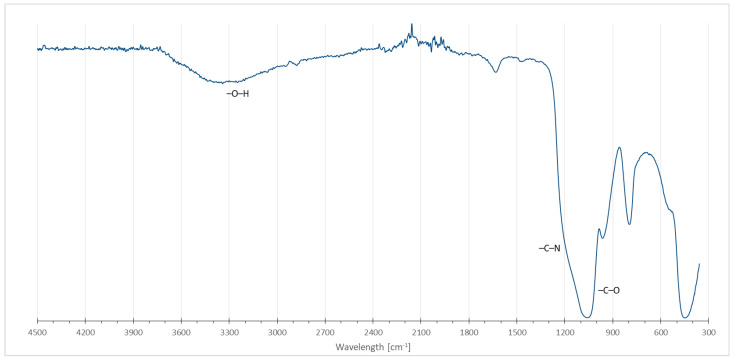
FTIR spectrum of MA-BDDE material.

Initial physicochemical analysis of the obtained material, performed using carbon-13 nuclear magnetic resonance (^13^C NMR) in solid state as well as Fourier-transformed infrared spectroscopy (FTIR), confirmed the presence of amine and ether groups within the structure. Figure 2 and Figure 3 show solid-state NMR and FTIR spectra, respectively.

The ^13^C NMR spectrum exhibits a strong peak at the value of 28.5 ppm, within a shift range where amine nitrogen C–N grouping feedback peaks occur, confirming the presence of these groups within the structure. The other strong signal, a doublet of 68 ppm and 74.2 ppm peaks, is an image characteristic of carbon–hydrogen and hydroxyl carbon–oxygen interactions. The most prominent peak visible on the ATR FTIR spectrum is a signal at a wavelength of 1051 cm^−1^, associated with the stretching of a single strong alkoxy carbon–oxygen C–O bond. Its width could be attributed to the presence of a second peak at around 1200 cm^−1^, indicative of an amine C–N bond. Lack of separation between these two peaks is likely the result of the polymeric nature of the material hindering the resolution of the measurement. A broad peak within the 2800–3700 cm^−1^ range is characteristic of a hydroxyl O–H stretch—the lack of any sharp peaks in this area might suggest a lack of nitrogen–hydrogen interactions, implying that virtually every methylamine nitrogen built into the structure of the MA-BDDE material is of the tertiary or quaternary ordinance.

### 2.2. HPLC Calibration

**Figure 4:**
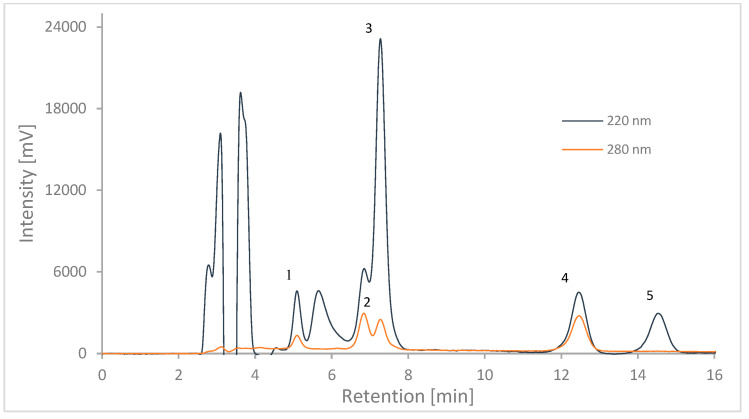
Chromatogram of 1 μg mL^−1^ pharmaceuticals solution. 1—aspirin; 2—ketoprofen; 3—naproxen; 4—diclofenac; 5—ibuprofen.

Five common NSAIDs were selected in order to evaluate the ability of the MA-BDDE sorbent to isolate organic ionic xenobiotics, namely aspirin (ACA), ketoprofen (KET), naproxen (NAP), diclofenac (DIC) and ibuprofen (IBP). Determination of the samples was carried out using liquid chromatography combined with detection in the visible and ultraviolet light spectrums (HPLC-UV/Vis system). Figure 4 shows a chromatogram obtained during the simultaneous analysis of all five analytes in a standard solution of methanol. Based on absorption spectra obtained for each of the pharmaceuticals, two detection channels were chosen for accurate analyte quantification. The primary channel was set for detection at a wavelength of 220 nm as it provided strong signals for all five compounds. Resolution between ketoprofen and naproxen (peaks 2 and 3 in Figure 4) was not satisfactory; therefore, a secondary channel was introduced for detection at 280 nm allowing for better separation of the signal from ketoprofen. For naproxen, the peak obtained from the primary channel was chosen for analysis, as this strong signal was unaffected by poor resolution, as is reflected by the good linearity value of its calibration curve, as shown in Table 1.

Calibration was performed by using five NSAID solutions at varying concentrations between 0.1 and 2 µg mL^−1^, and the experiments were performed in replicates of three. The coefficient of determination (R^2^) obtained for each analyte, as well as their respective detection (LOD) and quantitation limits (LOQ), are presented in Table 1. LOD and LOQ values were calculated in accordance with the guidelines found in *Analytical method validation: A brief review* [27] as LOD = 3.3 Sa/b and LOQ = 10 Sa/b, where Sa is the standard deviation of the calibration curve intercept, and b is the calibration curve slope.

### 2.3. Sorption Effectiveness

Extraction was carried out using the dSPE procedure due to its simplicity and ease of application combined with its effectiveness, as well as low sorbent and solvent expenditure. Table 2 shows the amount of each pharmaceutical absorbed by 1 g of the MA-BDDE sorbent and unmodified silica in simultaneous extraction. Calculation of the equilibrium sorption capacity Q was carried out using the following formula:

Q = (C_0_ − C_e_) × V/m (1)

where C_0_ is the initial concentration of the solution (μg mL^−1^), C_e_ is the equilibrium concentration (μg mL^−1^), V is solution volume (mL), and m is sorbent mass (mg). The obtained results clearly show the sorption capacity of the MA-BDDE sorbent has greatly improved over unmodified silica. The lower affinity of the material towards ketoprofen and ibuprofen is also easily noticeable.

**Figure 5:**
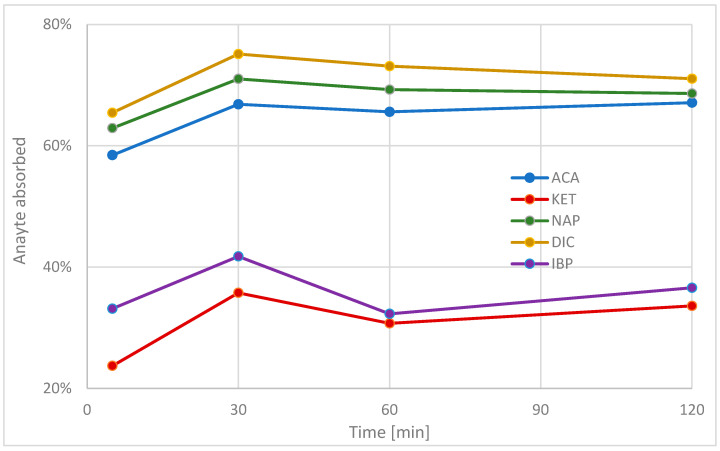
NSAID sorption effectiveness over time.

Initial analysis of the simultaneous extraction process from a mixed solution of 1 μg mL^−1^ reveals that around the 30 min mark, its effectiveness is the highest, while carrying the process past 60 min no longer brings any significant change to the NSAID content in the solution (Figure 5).

Desorption from the surface of the MA-BDDE sorbent was carried out using 1% acetic acid solution in methanol and produced very good recovery values (Table 3). In comparison, pure methanol proved to be an inadequate solvent for this purpose. It was noted, however, that during chromatographic analysis, the presence of acetic acid in the injected volume caused the signal corresponding to formic acid present in the mobile phase to shift slightly towards lower values, at times hindering the determination of the amount of aspirin in the sample.

### 2.4. Environmental Samples

For the purpose of evaluating the synthesized sorbent and the developed analytical procedure, samples were taken from two rivers: Vistula, a major river in Poland, and its tributary, Brda. Extraction and analysis were performed as described in Section 4 of this article. The results, as shown in Table 4, were unremarkable: while traces of some of the analyzed NSAIDs were found in Vistula River, no peak was observed on a chromatogram obtained from the Brda sample. The most likely explanation is the fact that Vistula is a great river and there are multiple large population centers located within its basin. Discharge from wastewater treatment plants accompanying these towns and cities flows to and eventually ends up in Vistula River waters. In comparison, Brda is a small river with just one city with a population of roughly 340,000 in its vicinity. As such, pharmaceutical contamination of its waters is bound to be much lower and, in this case, NSAID concentration was too low to be detected using our method.

In order to definitively confirm MA-BDDE efficiency, a final series of tests was conducted, where extraction was carried out as described on both river water samples as well as distilled and tap water samples, all of which were spiked with a mixed NSAID solution containing 100 µg of each pharmaceutical. Recoveries obtained in this experiment, shown in Table 5, are very similar to the high values displayed in Table 3, proving the high applicability of our sorbent in NSAID isolation from water.

## 3. Discussion

A novel material consisting of silica coated with dendrimeric copolymer MA-BDDE was successfully synthesized and applied for the extraction of five selected NSAIDs using the dSPE procedure for the first time. The initial characteristic of the material, using solid-state ^13^C NMR and FT-IR spectroscopy, strongly suggests that both amine and ether groups originating from co-monomers indeed constitute the structure of the obtained material. A remarkable increase in NSAID sorption capacity, when compared to unmodified silica used as a core of the dendrimer, further confirms successful preparation of the sorbent. Results obtained via the HPLC-UV/Vis system show the high effectiveness of the applied nanomaterial in isolating NSAIDs from solutions and water samples, especially in the case of aspirin, diclofenac and naproxen, confirming MA-BDDE’s potential for isolating these substances from natural matrixes.

Analysis of the progress of the adsorption process revealed that the equilibrium is reached after an hour into the procedure. This allowed for the development of a dSPE extraction procedure for the isolation of pharmaceuticals from water samples, which is described in detail in Section 4. Using this procedure, the sorbent was applied in the determination of NSAID traces present in natural water samples taken from two rivers. Samples taken from a major river showed ibuprofen and diclofenac contamination most likely originated from large population centers found upstream. Vistula’s expansive river basin is also rich in agriculture and animal husbandry, which could also contribute especially to diclofenac contamination. Samples from the Brda River did not contain detectable NSAID traces as it is a much smaller river and lacks significant sources of pharmaceutical contamination in its vicinity. Methanol and an acetic acid solution in methanol were tested as solvents used in desorption, and the latter was chosen due to its very good recovery values despite occasional issues with aspirin detection. Using this eluent after extraction from various water samples spiked with NSAIDs allowed for the recovery of over 72% of the adsorbed analytes. 

The performance of the entire analytical procedure developed for this study was compared with results presented in several recently published articles focused on the determination of NSAIDs in water samples from the perspective of the sensitivity of the employed analytical method, as shown in Table 6. As expected, using methods based on mass spectrometry detection, especially coupled with gas chromatography separation, allowed for the detection of much lower concentrations of analytes [28,29,30]. It is notable that Alinezhad et al. [31] presented results of a study on NSAID determination in water using high-pressure liquid chromatography followed by detection in ultraviolet light with exceptionally good validation parameters obtained. Modification of polyamidoamine dendrimer with magnetic Fe_3_O_4_ nanoparticles allowed for the employment of a magnetic solid-phase extraction procedure (MSPE). When combined with HPLC-UV detection, it resulted in an analytical method achieving sensitivity values easily comparable to those displayed in studies performing GC-MS analyses. Chen et al. [32], who also reported NSAID detection using a standard HPLC-UV system, presented results showing higher LOD values, which were very close to those obtained by Al-Khateeb and Dahas [33] using a similar detection method while also employing buffered superheated water in an HPLC mobile phase in order to improve mass transfer and analyte diffusion and therefore enhance separation. The displayed sensitivities of these two methods are only slightly higher and still comparable to the parameters of our developed procedure.

## 4. Materials and Methods

Methylamine (40% aqueous solution) and 1,4-butanediol diglycidyl ether (95%), as well as Silica Gel 60, were provided by Merck KGaA, Darmstadt, Germany. For the purposes of sorbent synthesis, following aqueous solutions were prepared: MA + BDDE (2.8% and 7.2%, respectively), MA (4%) and BDDE (10%). Aspirin (acetic salicyl acid), diclofenac (2-[(2,6-dichlorophenyl)amino]benzeneacetic acid sodium salt), ketoprofen (2-(3-benzoylphenyl)propionic acid), naproxen ((*S*)-(+)-2-(6-methoxy-2-naphthyl)propionic acid) and ibuprofen ((±)-2-(4-isobutylphenyl)propanoic acid) are certified pharmaceutical standards for analysis and were supplied by Merck KGaA, Germany. Pharmaceutical stock solutions in methanol at concentration of 100 mg L^−1^ were prepared and stored in darkness. Methanoic acid (80%) and methanol (HPLC-grade) were provided by Merck KGaA, Germany, and acetonitrile (methyl cyanide; HPLC-grade) was obtained from Honeywell International Inc., Charlotte, NC, USA. Acetic acid (99.5%) was purchased from CHEMPUR.

In order to coat silica with a binding layer of MA-BDDE copolymer, 5 g of silica gel was stirred for 30 min with the MA + BDDE solution at 65 °C. The material was then filtered, washed with deionized water and finally dried. The next two steps consisted of repeating the same procedure, using MA and BDDE solutions (in this order) instead of MA + BDDE solution, to obtain branching co-monomer layers, forming the first layer of dendrimeric MA-BDDE copolymer. Steps involving the use of MA and BDDE solutions were repeated four more times, resulting in five layers of dendrimerically branching MA-BDDE copolymer coating a silica core, which were proven to exhibit the best sorptive properties [34].

Initial physicochemical characterization of the obtained material consisted of infrared spectroscopic analysis with the help of a Bruker Alpha-P Attenuated Total Reflectance (ATR) Fourier Transform InfraRed (FT-IR) spectrometer, as well as carbon-13 nuclear magnetic resonance (^13^C-NMR) analysis in solid state using Bruker Ascend III 400 MHz (9,4T) spectrometer with an Avance III HD console.

Analyte extraction with MA-BDDE sorbent was carried out utilizing dSPE procedure. Around 5 mg of the sorbent and 15 mL of analyte solution in methanol were placed in a polypropylene centrifugal container that was then shaken at 80 RPM in order to disperse the sorbent within the solution volume. After each dispersion step, containers were placed in a centrifuge and rotated for 20 min at a speed of 4000 RPM. After centrifugation, 100 μL of supernatant was taken out of the container, out of which 20 µL was injected into HPLC apparatus for analysis.

After extraction, sorbent was filtered from the sample and briefly washed with methanol. It was then moved to another centrifugal container, where 10 mL of 1% acetic acid solution in methanol was subsequently added. The container was then shaken at a speed of 80 RPM for half an hour and centrifuged. After centrifugation, 100 μL of supernatant was taken for the purpose of performing HPLC analysis.

Surface water samples taken from Brda and Vistula rivers were first filtrated in order to remove any suspended particles. A total of 100 mL of the filtrate was then placed in flasks with 10 mg of MA-BDDE sorbent and stirred for half an hour. When the extraction phase was finished, the desorption procedure was carried out as described above.

Simultaneous analyte detection was conducted using a SHIMADZU Prominence HPLC-UV/Vis system. Mobile phase consisted of acetonitrile (40% *v*/*v*), methanol (30% *v*/*v*) and 0.5% formic acid aqueous solution (30% *v*/*v*). Separation was carried out with mobile phase flow of 0.5 mL min^−1^ in reverse phase, using Supelco Discovery HS C18 column with dimensions of 15 cm × 4.6 mm × 5 µm at 30 °C. Detection wavelengths of 220 and 280 nm were chosen based on absorption spectra of each analyzed pharmaceutical compound obtained with JENWAY 7315 spectrophotometer.

## 5. Conclusions

A novel material based on silica coated with a dendrimeric copolymer of methylamine (MA) and 1,4-butanediol diglycidyl ether (BDDE) was successfully synthesized for the first time. The initial characteristic of the material, labeled MA-BDDE, using solid-state ^13^C NMR and FTIR spectroscopy, confirms the presence of both amine and ether groups originating from co-monomers within the structure. The material was applied as a sorbent in a dispersive solid-phase extraction procedure of select pharmaceuticals from solutions and water samples, showing a good ability to isolate these compounds from natural matrixes. Coupling it with a standard HPLC-UV/Vis detection system created an effective analytical method with sensitivity comparable to most reported methods employing a similar methodology. The utilization of an acetic acid solution for desorption yielded high recovery values.

Small quantities of organic solvents that are required translate directly into equally small volumes of waste produced in the course of the analysis. The entire analytical procedure, from extraction to chromatographic separation and finally to detection, is extremely simple, as well as fast and effective, and does not rely on highly expensive or complex equipment. The liquid chromatograph combined with a UV/Vis detection system used in this study could be considered a standard apparatus that can be found in virtually any analytical laboratory today. As such, our method stands as a valid alternative for NSAID determination in natural water samples.

## Figures and Tables

**Table 1:** Validation parameters for pharmaceutical HPLC-UV/Vis analysis.

Analyte	R^2^	LOD [ng mL^−1^]	LOQ [ng mL^−1^]
Aspirin	0.9936	131	396
Ketoprofen	0.9744	265	802
Naproxen	0.9952	114	346
Diclofenac	0.9985	63	191
Ibuprofen	0.9970	89	270

**Table 2:** Sorption capacity of selected pharmaceuticals shown for silica modified with MA-BDDE dendrimer and unmodified silica.

Analyte	Sorption Capacity [mg g^−1^]	
	MA-BDDE	Silica Gel 60
Aspirin	2.005	0.08
Ketoprofen	1.073	0.11
Naproxen	2.131	0.11
Diclofenac	2.253	0.10
Ibuprofen	1.253	0.06

**Table 3:** Recovery values obtained using methanol and acetic acid solutions as solvents in desorption process.

Analyte	Recovery (%) (RSD (%); *n* = 3)	
	Methanol	1% HAc *
Aspirin	22 (8.5)	n.d.
Ketoprofen	4 (5.8)	96 (4.3)
Naproxen	9 (6.8)	103 (14.3)
Diclofenac	12 (6.1)	88 (9.7)
Ibuprofen	26 (11.0)	77 (10.1)

* 1% acetic acid solution in methanol.

**Table 4:** NSAID concentrations found in river samples.

Analyte	Concentration (ng mL^−1^)	
	Vistula	Brda
Aspirin	n.d.	n.d.
Ketoprofen	n.d.	n.d.
Naproxen	n.d.	n.d.
Diclofenac	32.7	n.d.
Ibuprofen	31.6	n.d.

n.d.: not determinated.

**Table 5:** NSAID % recovery and standard deviation values (%, *n* = 3) from spiked water samples.

	Brda River	Vistula River	Distilled Water	Tap Water
Aspirin	80 (13.8)	n.d.	n.d.	92 (5.3)
Ketoprofen	92 (8.1)	88 (3.3)	99 (3.7)	99 (6.3)
Naproxen	98 (8.7)	93 (4.8)	88 (7.8)	96 (4.5)
Diclofenac	96 (6.1)	101 (6.0)	93 (6.3)	92 (5.2)
Ibuprofen	72 (9.3)	86 (10.1)	79 (7.6)	83 (9.3)

n.d.: not determinated.

**Table 6:** Comparison of the developed methods’ performance with recently reported data.

Analyte	Analytical Method	LOD (ng mL^−1^)	Linear Range (ng mL^−1^)	Reference
Diclofenac, ibuprofen, ketoprofen, naproxen	FPSE ^a^-GC-MS	0.8–5	0.005–0.5	[28]
Phenlacetin, meloxicam, naproxen, diclofenac, carprofen	SPE-UHPLC-MS/MS	0.019–0.041	0.02–5	[29]
Piroxcam, meloxicam, ketoprofen, naproxen, diclofenac, indomethacin, mefenamic acid, tolfenamic acid	MSPE ^b^-UHPLC-MS/MS	0.003–0.06	0.02–25	[30]
Naproxen, diclofenac, ibuprofen	MSPE-HPLC-UV	0.05–0.08	0.15–500	[31]
Ketoprofen, naproxen, diclofenac, ibuprofen	SPE-HPLC-UV	2–32	5–500,000	[33]
Ibuprofen, diclofenac, ketoprofen, 4-CAA ^c^, clofibric acid	dSPE-HPLC-UV	2.9–21.4	10–10,000	[32]
Aspirin, diclofenac, ibuprofen, ketoprofen, naproxen	dSPE-HPLC-UV/Vis	63–265	100–2000	present study

^a^—fabric-phase sorptive extraction; ^b^—magnetic solid-phase extraction; ^c^—4-chlorophenoxyacetic acid.

## Data Availability

All data generated during this research are presented within the article.
